# Assessing the Readability of Online Health Information for Colonoscopy — Analysis of Articles in 22 European Languages

**DOI:** 10.1007/s13187-023-02344-2

**Published:** 2023-07-26

**Authors:** Tomasz Skrzypczak, Michał Mamak

**Affiliations:** 1 Wroclaw Medical University, Wybrzeże L. Pasteura 1, 50-367 Wrocław, Poland; 2 University Hospital in Wroclaw, Borowska 213, 50-556 Wrocław, Poland

**Keywords:** Colonoscopy, Colorectal prophylaxis, Health information, Internet content

## Abstract

Patients often search on the Internet information about different medical conditions and procedures. This study aimed to evaluate online health information on colonoscopy, focusing on quantity and comprehensibility of internet resources dedicated to the colonoscopy. This information could be used by European Union (EU) colorectal cancer (CRC) screening providers to address patient’s unfilled educational needs, fear of colonoscopy, and other barriers that deter from CRC screening. The term “colonoscopy” translated into 22 official EU languages was searched using the Google search engine. For each translation, generated list of websites was assessed with Google Translate. The first 50 websites in each language were assessed for suitability. Records in other languages were excluded. Included websites were free, focused on patient education, and did not have password. Readability assessments were performed with Lix score. A total of 588 websites in Bulgarian, Croatian, Czech, Danish, Dutch, English, Estonian, Finnish, French, German, Greek, Hungarian, Italian, Latvian, Lithuanian, Polish, Portuguese, Romanian, Slovak, Slovenian, Spanish, and Swedish were evaluated. The overall mean Lix score was 56 ± 8 and was classified as very hard to comprehend. There were significant differences in mean Lix scores across the included languages (*P*<.001). There was not significant correlation (*R*^2^ = 0.1, *P* = 0.142) between Lix score and number of search hits. Although there was a wealth of online patient information on colonoscopy, the comprehensibility of the available information is low. Physician guidance to reliable resources could increase patient’s willingness to undergo a screening colonoscopy.

## Introduction

Patients use the internet to learn about their medical problems and available treatment due to the user-friendliness, accessibility, and low cost of online health information [1]. The Internet is one of the most trusted sources of health information by the public [1]. Content presented in the internet is not regulated; significant quality variations were demonstrated on public websites dedicated to various health conditions [2]. Searches related to information about cancer have increased in recent years [1]. Colorectal cancer-related queries encompassed 13% of those searches [1]. Not only cancer patients but also physicians increasingly access online information to build on their expertise and supplement their own knowledge [1].

Colorectal cancer (CRC) is the second deadliest cancer in the European Union (EU) [3]. If detected early enough, CRC is both curable and preventable [3]. Most of current guidelines recommend colonoscopy as CRC screening gold standard [4]. Despite multiple programs established in the EU member states, only approximately 14% of EU citizens between 50 and 75 years old participate in population-based screening programs [3]. Lack of CRC awareness and understanding and unfilled patient’s educational needs contribute to low participation rates [3]. It was demonstrated that patients want to educate themselves with online patient electronic materials [5]. With use of Google — the most popular search engine, patients query search term aimed to generate list of websites that would potentially resolve their doubts [5].

The Google-found patient online information is a mix of articles dedicated to health professional, patients, journalists, pharmacist, and public [6]. Patient’s and public dedicated articles could overestimate readers’ health literacy by using medical words and phrases that exceed patients’ capacity for understanding [6]. Readability is defined as “the ease with which written materials are read” and is critically important factor in assessing how well a patient-focused resource may be comprehended, with increased readability being associated with increased comprehension [6]. It could not be excluded that low readability of patient electronic materials related to colonoscopy deters patients from CRC screening programs’ attendance. Colonoscopy is invasive and intimate procedure. Lack of understanding the procedure course and preparation for the colonoscopy and potential complications increase perceived patient’s fear. This was identified as a critical barrier that deter patients from prophylactic colonoscopy [3].

There were multiple studies focused on patient electronic CRC-related material’s readability [2, 7–13]. No study was found that examined readability of Google-searched electronic materials dedicated to colonoscopy written in European languages. Previous studies were focused mainly on materials written in English, which do not represent the spectrum of European patients. The main aim of this study was to assess readability of Google-searched patient electronic materials dedicated to colonoscopy written in the EU languages with validated measure. The secondary aim was to evaluate prevalence of those materials in the included languages. Finally, correlation between popularity and readability of those materials was examined.

## Methods

### Search Strategy

Search term “colonoscopy” was translated with Google Translate services to official languages of the EU. Those terms were queried in the Google search engine. Language of each result from generated list was assessed with Google Translate. Results in other languages than searched term were excluded. The first 50 search results in the searched term language were recorded. Previous studies demonstrated that internet users do not read beyond the 50 hits [14]. Online forums, advertisements, personal blogs, videos, and scientific articles were excluded. A website was classified as an advertisement if primarily contained promotional material for a specific drug, clinician, and medical center and/or did not have a focus on patient education [1]. Online electronic materials dedicated to other procedures (i.e., colposcopy, gastroscopy, sigmoidoscopy), natural remedies, other cancers (i.e., cervical cancer), vaccinations (i.e., COVID-19), prevention services, and personalized colorectal centers also were excluded. Included websites were written in searched term language, were not password protected, were free to the public, and had focus on patient education. The EU has 24 official languages: Bulgarian, Croatian, Czech, Danish, Dutch, English, Estonian, Finnish, French, German, Greek, Hungarian, Irish, Italian, Latvian, Lithuanian, Maltese, Polish, Portuguese, Romanian, Slovak, Slovenian, Spanish, and Swedish [15]. In this study, Maltese and Irish were excluded due to very low search yield (64 and 95 hits, respectively) and negligible prevalence (0 and 2 included websites, respectively).

### Readability Assessment

Included articles were evaluated with Lix formula, a validated readability measure [16]. Unlike other measures (i.e., the Gunning Fogg Index), Lix was proved to be reliable readability measure across several languages (Swedish, Danish, English, French, German, Finnish, Italian, Spanish, Portuguese) [17]. It is considered by scientific community to be a reliable readability measure for all European languages [16, 17]. Apart from being easy to calculate and interpret, it bypasses issues with syllabification, which makes it suitable for even such complex languages like Chinese and Arabic [16]. Website text was copied into the Microsoft Word and all extraneous text (i.e., hyperlinks, affiliations, figures, legends, adverts, disclaimers, author information, copyright notices, and author information) was removed. Save as Plain Text function was utilized. Then, relevant language of the analyzed website was selected. Spelling & Grammar was checked and corrected by Microsoft Word. Then, text was copied to https://haubergs.com/rix online Lix calculator. Lix score, number of sentences, number of words, and average number of words in one sentence were recorded. When interpreting Lix scores, scale proposed by *Anderson* was applied [17]. Text with score < 20 was classified as very easy to comprehend, < 30 easy, < 40 little hard, < 50 hard, and < 60 very hard to comprehend [17].

### Statistical Analyses

Mean LiX scores, number of sentences, and number of words were compared across all analyzed languages using analysis of variance (ANOVA). To examine correlation between number of hits and mean Lix score of analyzed articles, univariate linear regression was utilized. *P* value equal or less than 0.05 was considered statistically significant. Analyses were conducted using JASP version 0.17.1 (JASP Team, University of Amsterdam). In this study, Microsoft Word version 16.59 (Redmont, USA) was utilized.

## Results

### Popularity

In general, 588 websites were included in the analysis. The highest numbers of articles were in German (41 articles, 7%) and French (41 articles, 7%). Bulgarian was the least popular language with only 8 (1.4%) articles included. The highest number of hits was revealed for Portuguese (11.3 million), Spanish (10.5 million), and English (6.15 million). The lowest number of Google search hits was observed for Hungarian (38,200). Included number of websites, search hits, and searched queries are presented in Table 1.

**Table 1:** Number of hits and included websites per search term

Language	Search term	Total # of hits	Included websites *n* (%)
Bulgarian	колоноскопия	4,270,000	8(16)
Croatian	kolonoskopija	175,000	26(52)
Czech	kolonoskopie	571,000	28(56)
Danish	koloskopi	215,000	35(70)
Dutch	colonoscopie	310,000	25(50)
English	colonoscopy	61,500,000	37(74)
Estonian	kolonoskoopia	4,480,000	17(34)
Finnish	Kolonoskopia	4,480,000	25(50)
French	coloscopie	2,170,000	41(82)
German	Darmspiegelung	3,040,000	41(82)
Greek	Κολονοσκόπηση	175,000	22(44)
Hungarian	kolonoszkópia	38,200	20(40)
Italian	colonscopia	184,000	35(70)
Latvian	kolonoskopija	320,000	19(38)
Lithuanian	kolonoskopija	210,000	23(46)
Polish	kolonoskopia	4,590,000	38(76)
Portuguese	colonoscopia	11,300,000	30(60)
Romanian	colonoscopie	352,000	24(48)
Slovak	Kolonoskopia	4,790,000	19(38)
Slovenian	kolonoskopija	232,000	20(40)
Spanish	colonoscopia	10,500,000	35(70)
Swedish	koloskopi	202,000	20(40)

### Readability Assessment

Overall mean values for analyzed articles were 56 ± 8 for Lix score, 59 ± 16 for number of sentences, 859 ± 262 for number of words, and 15 ± 3 for number of words per sentence. All differences between languages were statistically significant (all *P*<.001). Values for included languages are presented in Table 2.

**Table 2:** Readability of colonoscopy-related online patient information in 22 European languages

Language	Lix score	#sentences	#words	#words/sentence
Bulgarian	64±4	78±42	1011±478	14±2
Croatian	61±6	39±22	575±381	15±4
Czech	54±6	46±25	639±329	15±4
Danish	44±6	48±41	712±634	15±3
Dutch	43±5	72±61	935±690	14±2
English	40±5	75±46	1221±797	17±3
Estonian	59±6	52±31	515±282	11±2
Finnish	65±6	39±26	408±234	11±2
French	50±5	68±40	1177±707	17±4
German	49±5	79±66	1013±852	13±3
Greek	58±5	38±20	754±372	21±4
Hungarian	66±4	47±20	691±289	15±2
Italian	62±4	65±47	1241±783	20±3
Latvian	62±4	46±26	663±374	15±4
Lithuanian	64±6	42±30	499±355	12±3
Polish	63±6	68±45	968±587	15±3
Portuguese	52±6	52±29	912±434	18±4
Romanian	61±6	56±37	1128±768	21±4
Slovak	58±8	80±133	767±456	14±4
Slovenian	59±5	43±28	703±485	16±3
Spanish	48±5	74±54	1177±757	16±3
Swedish	43±8	81±66	1182±1162	14±3

Data were presented as mean ± SD. # stands for number of sentences. Differences between Lix score, number of sentences, number of words, and number of words/sentence were statistically significant. All *P*<.001

**Fig. 1:**
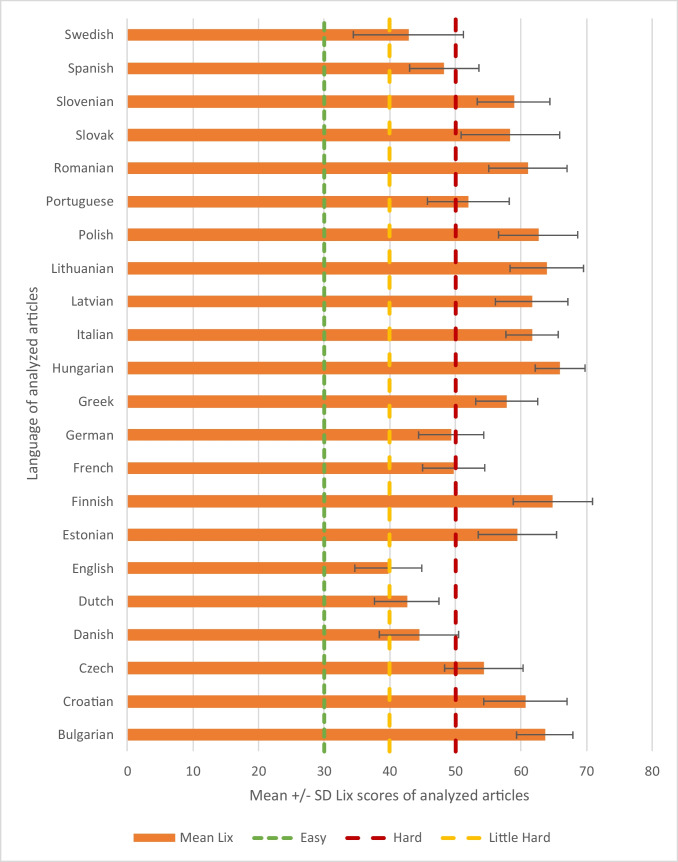
Readability of colonoscopy-related online patients electronic materials in 22 European languages. Mean Lix scored for websites generated from “colonoscopy” search in 22 official EU languages. Easy refers to Lix score <30 and classifies text as easy to comprehend. Little hard refers to Lix score <40 and classifies text as little hard to comprehend. Hard refers to Lix score <50 and classifies text as hard to comprehend

Articles with the highest words/sentence ratios were found for Romanian (21±4) and Greek (21±4). The lowest words/sentence ratios were found for Finnish (11±2) and Estonian (11±2). Articles with the highest average numbers of sentences were found in Swedish (81±66), Slovak (80±133), and Bulgarian (78±42). The opposite was found for articles in Greek (38±20), Croatian (39±22), and Finnish (39±26). The highest average word numbers per article were found for Italian (1241±783), Swedish (1182±1162), French (1177±707), Spanish (1177±757), and Romanian (1128±768). The lowest mean number of words per article was found for Finnish (408±234). Mean Lix scores for analyzed languages are presented in Figure 1.

The most comprehendible materials were found in English (40±5), Dutch (43±5), Danish (44±6), and Swedish (43±8). The most difficult articles to comprehend were found in Hungarian (66±4), Finnish (65±6), Bulgarian (64±4), and Lithuanian (64±6). No articles in included languages had Lix score <30 or <40 and could be classified as easy or little hard to comprehend. Articles in Swedish, Spanish, German, English, Dutch, and Danish were classified as hard to comprehend. Articles in remaining included languages were classified as very hard to comprehend.

### Readability and Prevalence

To examine correlation between readability of online patient’s electronic materials and number of Google search hits, univariate linear regression was conducted. No significant correlation was revealed (*R*^2^ = 0.1, *P* = 0.142).

## Discussion

Our results suggest that, although the internet provides a large amount of information on colonoscopy, the comprehensibility of available materials is not optimal. This is highlighted by the fact that materials in any included languages were classified as easy to comprehend. Materials in all analyzed languages had at least “little hard” to comprehend category. Presented results also revealed that quantity did not equate to readability in the context of online health information. The number of hits did not correlate with mean Lix scores.

Overall mean Lix score was 56 ± 8 which corresponded to college grade level [18]. In 2021, only 22.1% of EU population aged 55–74 had completed tertiary education [19]. These facts could mean that only one out of five patients that fall within screening colonoscopy inclusion criteria is able to understand colonoscopy-related information found on the Internet. Lack of colonoscopy course and preparation and complications understanding may increase perceived fear that deters from the procedure. It was demonstrated that patients seek answers for the questions that were reluctant to disclose to the physician on the Internet [5]. Difficult to comprehend articles could cause further misunderstanding, confuse patients, and finally deter even those who were willing to undergo colonoscopy. In all 22 included official EU languages, low comprehensibility of online materials was revealed, which is a potential challenge for CRC screening providers. It seems reasonable to assume that introduction of comprehensible colonoscopy-related educational campaign with online patient materials would potentially increase CRC screening participation rates. Google search engine offers a web promotion [20]. Promoted websites are positioned at the top of search results, which makes them the most likely to be visited by the Internet user [20]. Promotion of medically verified, easy to comprehend website that is attractive for the Internet user could be potentially helpful. Top-searched websites, presented in all EU official languages, have a potential to resolve doubts of patients and encourage them to attend the CRC screening program.

This observed gap in online health information was also demonstrated for other CRC search terms as well. It was revealed that Google-searched websites with search terms “colorectal cancer,” “colon,” “rectal cancer,” and “anal cancer” had very low readability of information [1]. In accordance with our results, large abundance of searched information was not correlated with readability [1]. In a study analyzing the quality and readability of websites pertaining to CRC screening in the USA, only 10% of the reviewed websites were rated as “high quality” [1, 2]. Similarly, systematic review that evaluated internet patient information on CRC surgery revealed the gaps in the information provided to patients, specifically risk and benefits of surgery, postoperative complications, and a detailed description of the surgical procedures [1, 21]. All of these suggest an overarching need for improved quality health resources related to CRC.

### Limitations

The Google search results are dynamic and may vary based on the geographic location of the search, date, and time. Presented results could be influenced by the location and date where the search was performed. This study was conducted in Poland and Google; search results were evaluated between 1st and 20th March 2023. Similarly, selection of Google as the search engine could bias the results. Furthermore, the websites were all evaluated with the Lix score. Lix was originally designed to evaluate readability of newspapers articles written in Swedish [17]. It was validated on multiple languages as reliable measure of readability (Swedish, Danish, English, French, German, Finnish, Italian, Spanish, Portuguese) [16, 17, 22, 23]. No studies were found that validated Lix for other included languages. However, Lix is considered by scientific community to be reliable readability measure for all European languages [16, 17]. Levels of comprehensibility applied in our study were designed for Danish, Swedish, Norwegian, and Dutch [16, 17, 22, 23]. It cannot be excluded that different cutoffs would be suitable for other included EU languages. Excluded websites (i.e., advertisements) were not assessed for readability, but they are also some sources of information for patients. Other social media platforms were not evaluated, such as Twitter, where a lot of online health information is also disseminated [1]. However, this was outside the intended scope of the study and could be a potential future area of research.

## Conclusions

Although the Internet provides plenty of free, easily accessible information on colonoscopy, the comprehensibility of the presented information is low. Only one out of five patients that fall within screening colonoscopy inclusion criteria can understand colonoscopy-related information found on the Internet. Presented findings suggest a great need for both EU and regional CRC screening providers’ involvement in creation of high-quality, online information for patient education about colonoscopy. Multilingual, attractive for patients, reliable, and readable website dedicated to colonoscopy should be designed and promoted by responsible EU institutions. Google search engine optimization for maximal accessibility of validated websites would also be beneficial. This is particularly important as number of “colonoscopy” hits highlighted that this procedure is keen interest to the public.

## Data Availability

Data are available upon reasonable request to the correspoding author.
